# Antihypertensive Effects of Two Novel Angiotensin I-Converting Enzyme (ACE) Inhibitory Peptides from *Gracilariopsis lemaneiformis* (Rhodophyta) in Spontaneously Hypertensive Rats (SHRs)

**DOI:** 10.3390/md16090299

**Published:** 2018-08-27

**Authors:** Zhenzhen Deng, Yingjuan Liu, Jing Wang, Suhuang Wu, Lihua Geng, Zhenghong Sui, Quanbin Zhang

**Affiliations:** 1 CAS Key Laboratory of Experimental Marine Biology, Institute of Oceanology, Chinese Academy of Sciences, Qingdao 266071, China; dengzhenzhen16@mails.ucas.ac.cn (Z.D.); liuyingjuan829@163.com (Y.L.); jingwang@qdio.ac.cn (J.W.); wusuhuang16@mails.ucas.ac.cn (S.W.); genglihua13@mails.ucas.ac.cn (L.G.); 2 Lab for Marine Biology and Biotechnology, Qingdao National Lab for Marine Sci. & Tech, Qingdao 266071, China; 3 University of Chinese Academy of Sciences, Beijing 100049, China; 4 Center for Ocean Mega-Science, Chinese Academy of Sciences, 7 Nanhai Road, Qingdao 266071, China; 5 College of Marine Life Sciences, Ocean University of China, Qingdao 266071, China

**Keywords:** *Gracilariopsis lemaneiformis*, ACE-inhibitory activity, peptide, molecular docking, SHRs

## Abstract

A variety of biologically active products have been isolated from *Gracilariopsis lemaneiformis*. In the present study, two novel angiotensin-converting enzyme (ACE) inhibitory peptides, FQIN [M(O)] CILR, and TGAPCR, were screened and identified from *G. lemaneiformis* protein hydrolysates by LC-MS/MS. The IC50 values of FQIN [M(O)] CILR and TGAPCR were 9.64 ± 0.36 μM and 23.94 ± 0.82 μM, respectively. In the stability study, both peptides showed stabilities of pH, temperature, simulated gastrointestinal digestion, and ACE hydrolysis. The Lineweaver–Burk plot showed that the two peptides were noncompetitive inhibitors of ACE. Molecular docking simulated the intermolecular interactions of two peptides and ACE, and the two peptides formed hydrogen bonds with the active pockets of ACE. However, FQIN [M(O)] CILR was more closely linked to the active pockets of ACE, thereby exerting better ACE inhibition. Spontaneously hypertensive rats (SHRs) were studied with an oral dose of 10 mg/kg body weight. Both peptides reduced systolic blood pressure (SBP) and diastolic blood pressure (DBP) in SHRs, of which FQIN [M(O)] CILR was able to reduce the systolic blood pressure by 34 mmHg (SBP) (*p* < 0.05). Therefore, FQIN [M(O)] CILR was an excellent ACE inhibitory peptide.

## 1. Introduction

Hypertension (high blood pressure) refers to the main feature of increased body circulation arterial blood pressure (systolic blood pressure ≥140 mmHg and diastolic blood pressure ≥90 mmHg), which is associated with increased mortality of cardiovascular disease and diabetes [1]. The WHO reported that more than 17.5 million people have died each year as a result of cardiovascular disease. Hypertension has become the leading global cause of death [2]. Human blood pressure is mainly regulated by the systemic renin-angiotensin system (RAS) and the kallikrein-bradykinin system (KKS). The role of the renin-angiotensin system in regulating blood pressure relies on the proteolytic cascade of two enzymes: renin (EC 3.4.23.15), an aspartyl protease secreted into the blood from the liver, and angiotensin I-converting enzyme (ACE; EC 3.4.15.1), a carboxypeptidase present on the external surface of endothelial cells of the lung [3]. Renin hydrolyzes the angiotensinogen (α-globulin) secreted by the liver to the inactive angiotensin I. Next, angiotensin I is hydrolyzed by ACE to angiotensin II, which then binds to angiotensin receptors and causes vasoconstriction [4]. In addition, ACE can inactivate bradykinin, a nonapeptide with cardiac protection. When bradykinin binds to β-receptor, a series of reactions are generated and causes the intracellular Ca^2+^ levels to increase significantly. Ca^2+^ stimulates the nitric oxide synthase (eNOS) reaction to produce NO, which results in vasodilation and reduced blood pressure [5]. Angiotensin I-converting enzyme is a ubiquitous Zn^2+^-dependent carboxydiopeptidase in mammalian tissues that regulates the renin-angiotensin system (RAS) and the kallikrein-bradykinin system (KKS) and is considered useful therapeutic target for treating hypertension [6,7,8]. The common ACE inhibitors on the market are mostly “Prils”, such as captopril, enalapril, lisinopril, fosinopril and the like. While these drugs can produce a strong antihypertensive effect, they may also cause a series of side effects such as cough, rash, nausea, acute renal failure, and proteinuria. These drugs are believed to reduce patients’ compliance, leading eventually to exacerbated disease conditions, and increased health care costs [9]. For this reason, researchers are increasingly interested in developing natural ACE inhibitory peptides with low-toxicity that could replace or complement antihypertensive drugs [10]. Food-derived active peptides not only produce the same activity as ACE inhibitor drugs but are also safer in terms of having low toxicity due to their natural source. Components of proteins in food are containing sequences of bioactive peptides, which could exert a physiological effect in the body. These short chains of amino acids are inactive within the sequence of the parent protein, but can be released during gastrointestinal digestion, food processing, or fermentation [11]. It has been reported that ACE inhibitory peptides obtained from enzymatic, fermentative, or gastrointestinal digestion of foods exert significant antihypertensive effects in clinical and preclinical studies [12]. This demonstrates the significance and prospect of finding active ACE inhibitory peptides from healthy foods.

*Gracilariopsis lemaneiformis* is a red algae that is widely distributed in the marine environment and has important economic value [13]. *Gracilariopsis lemaneiformis* belongs to the family *Gracilariopsisceae* (Rhodophyta), in which a majority of the members are utilized as the main sources of the manufacture of agar [14]. *Gracilariopsis lemaneiformis* is rich in polysaccharides, protein, vitamins, and multiple minerals such as phosphorus, calcium, iodine, iron, zinc and magnesium [15]. Polysaccharides, phycoerythrin, agar and other active substances isolated from *G. lemaneiformis* have proven to show a range of biological activities such as antioxidation, antitumor, immune regulation and alcoholic liver injury protection [16,17,18,19]. There are few studies, however, investigating active peptides of *G. lemaneiformis*, particularly ACE inhibitory peptides. To date, ACE inhibitory peptides have been extracted from various types of marine algae, such as *Saccharina longicruris* [20], *Palmaria palmate* [21], *Pyropia columbina* [22], *Enteromorpha clathrata* [23], *Ulva rigida* C. [24], *Chlorella vulgaris* [25], and *Porphyra yezoensis* [26]. This demonstrates that marine algae are an important source of active peptides.

In this study, ACE inhibitory peptides were screened from *G. lemaneiformis* enzymatic hydrolyte. By searching the online database, the physicochemical properties of the peptides were investigated. The stabilities of such peptides against pH, temperature, gastrointestinal digestion and ACE hydrolysis were then assessed. Molecular docking was used to simulate the intermolecular interactions of peptides with ACE. Finally, the antihypertensive activities of the peptides were tested in vivo in spontaneously hypertensive rats (SHRs).

## 2. Results

### 2.1. Screening of the Active ACE Inhibitory Peptides.

Two ACE inhibitory peptides were screened and identified by LC-MS/MS; the sequences are FQIN [M(O)] CILR and TGAPCR. The result shows that the peptides are two novel ACE inhibitors with the IC50 value of 9.64 ± 0.36 μM and 23.94 ± 0.82 μM, respectively.

### 2.2. Properties of Peptides

The physicochemical properties of the peptide were obtained by online database search. Table 1 shows that the isoelectric points (PI) of FQIN [M(O)] CILR and TGAPCR are 8.61 and 8.55, respectively. Both peptides are non-toxic and can be used for animal studies. FQIN [M(O)] CILR has poor water solubility.

### 2.3. Stability Study for ACE Inhibitory Activity

**Figure 1:**
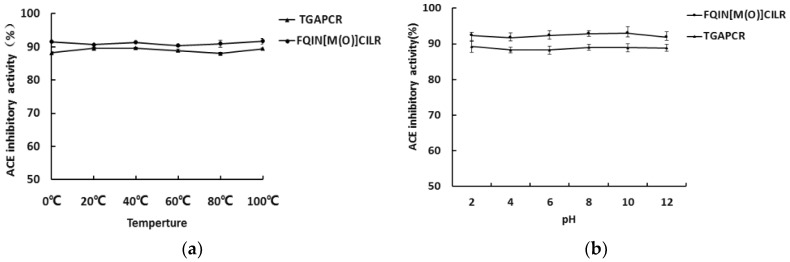
Temperature (**a**) and pH (**b**) stability of peptides FQIN [M(O)] CILR and TGAPCR. The concentration of peptides was 0.5 mg/mL; values represent mean ± SD (*n* = 3).

The pH and thermal stability of the peptides were measured. The results show that the peptides could maintain their ACE inhibitory activity after pH and heat treatment (Figure 1a,b). Both peptides show pH and thermal stability.

**Figure 2:**
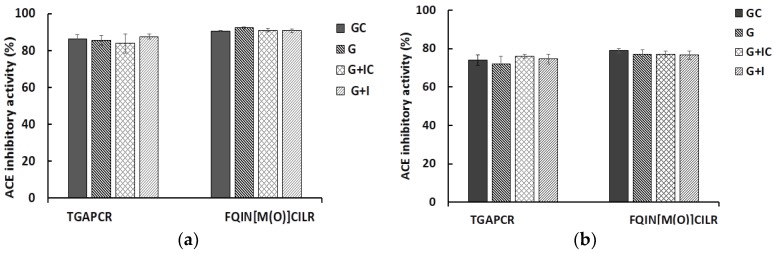
Stability of FQIN [M(O)] CILR and TGAPCR under simulated gastrointestinal digestion. The final concentrations of peptides were 0.5 mg/mL (**a**) and 0.1 mg/mL (**b**), respectively. GC, peptide sample and simulated gastric fluid without pepsin; G, peptide sample with gastric fluid with pepsin; G + IC, peptide sample and simulated gastrointestinal fluid without pepsin and pancreatin; G + I, peptide sample with gastrointestinal fluid with pepsin and pancreatin. Values represent mean ± SD (*n* = 3).

The stability of the peptides in the simulated gastric juice and intestinal fluid is shown in Figure 2. After digestion in simulated gastric juice and intestinal fluid containing the corresponding enzymes, the ACE inhibitory activity of the peptides did not display any significant changes. The results demonstrate that these peptides have gastrointestinal stability, and they may also produce antihypertensive effects in vivo.

### 2.4. Stability of Peptides against ACE

**Figure 3:**
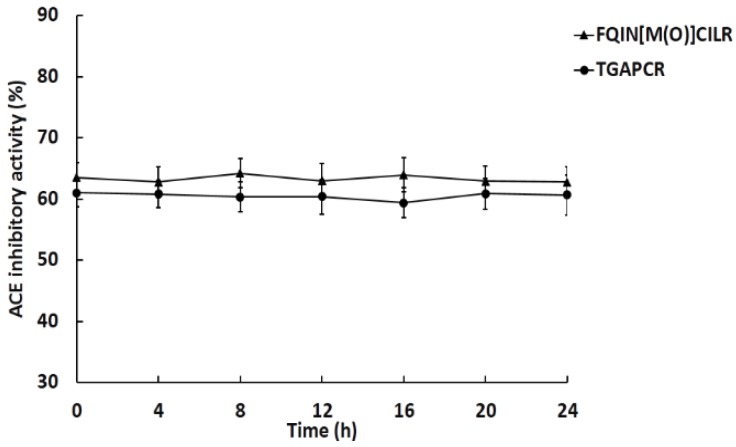
Stability of peptides against ACE. The concentration of peptides was 0.1 mg/mL; values represent mean ± SD (*n* = 3).

Figure 3 shows no significant changes of ACE inhibitory activity within incubation of ACE for 24 h. The peptides maintained stable ACE inhibitory activity, suggesting that they were not hydrolyzed by ACE.

### 2.5. Characterization of the Inhibition Pattern on ACE

**Figure 4:**
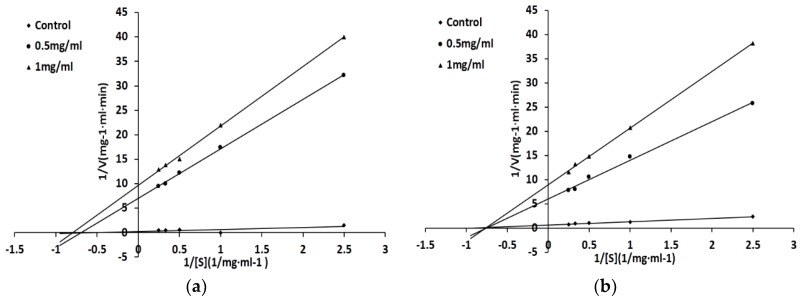
Lineweaver–Burk plots of ACE inhibited by the peptides. 1/V and 1/S represents the reciprocal of reaction velocity and substrate concentration, respectively. (**a**) FQIN [M(O)] CILR; (**b**) TGAPCR.

Based on the results of the Lineweaver–Burk plots, both FQIN [M(O)] CILR and TGAPCR are noncompetitive inhibitors of ACE. Table 2 shows the kinetic parameters of the two peptides binding to ACE. Combined with the results shown in Figure 4, the value of Km (2.26 ± 0.21 mM) is constant, which is an important characteristic of noncompetitive inhibition. In the noncompetitive inhibition pattern, the substrate and the inhibitory peptide bind to different parts of the enzyme. Thus, the apparent affinity of the substrate and the enzyme is a constant [27]. The Vmax (2.23 ± 0.67 mg^−1^·mL·min) decreases as the concentration of inhibitory peptide increases. This is due to the reduction of the apparent catalytic efficiency of ACE, which leads to the decrease of the enzymatic reaction efficiency [23]. At the same concentration, the Vmax of FQIN [M(O)] CILR is smaller than TGAPCR, indicating that FQIN [M(O)] CILR produce a better inhibitory activity than TGAPCR.

The Ki of FQIN [M(O)] CILR and TGAPCR are 0.71 ± 0.04 mM and 0.86 ± 0.06 mM, respectively. The Ki value indicates the affinity of ACE inhibitory peptide to ACE. The lower the inhibition constant (Ki), the higher the affinity of the inhibitors to ACE. The results show that FQIN [M(O)] CILR can produce better inhibitory activity than TGAPCR.

### 2.6. Molecular Docking

**Figure 5:**
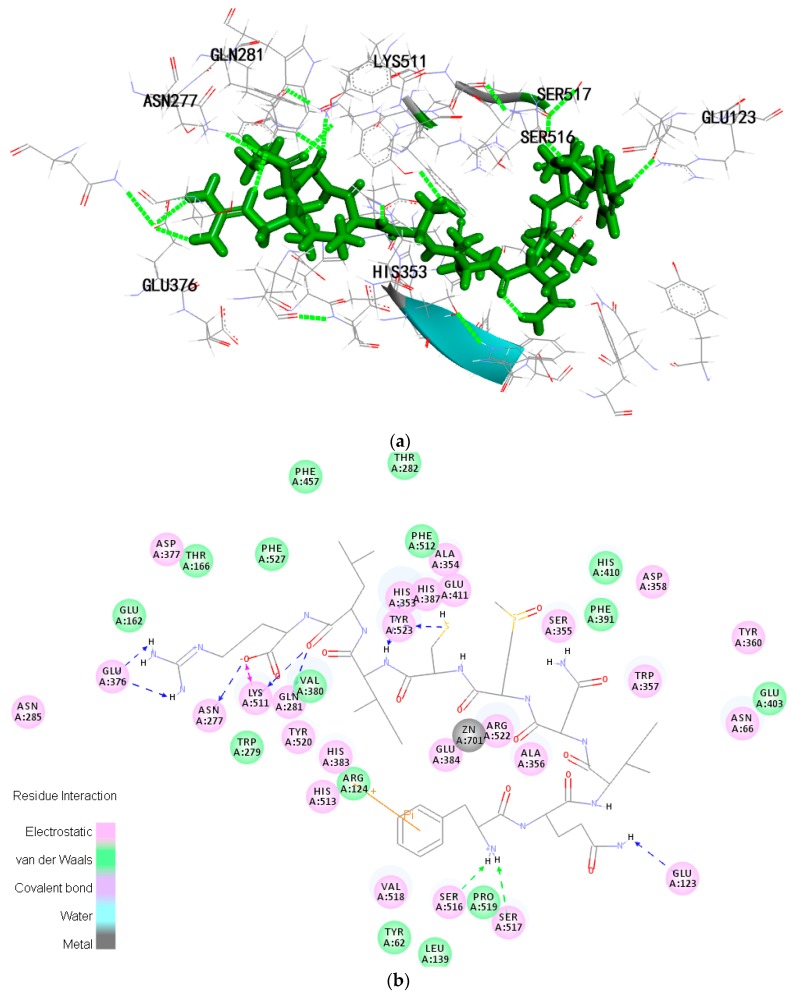

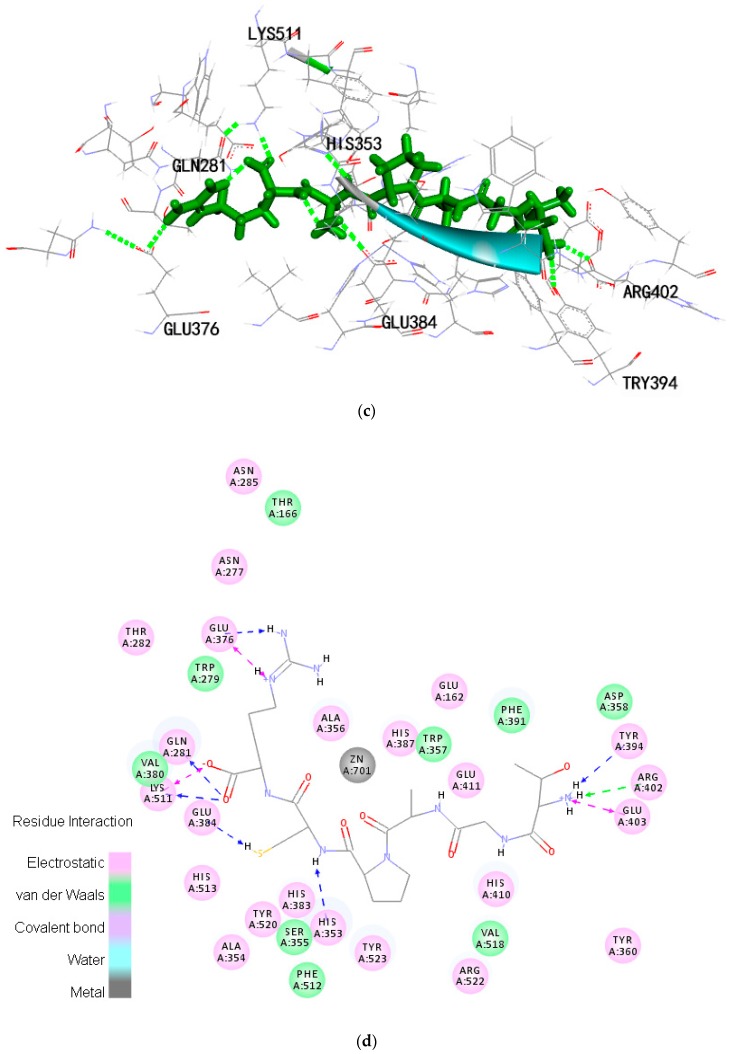
Molecular docking simulation of the peptides. (**a**,**b**) are local overview and two-dimensional (2D) diagram of FQIN [M(O)] CILR; (**c**,**d**) are local overview and two-dimensional (2D) diagram of TGAPCR.

CDOCKER was used to discover the ligand−receptor interaction mechanism. The -CDOCKER ENERGY and -CDOCKER INTERACTION ENERGY of FQIN [M(O)] CILR are 158.117 kcal·mol^−1^ and 105.509 kcal·mol^−1^, respectively. The -CDOCKER ENERGY and -CDOCKER INTERACTION ENERGY of TGAPCR are 90.4226 kcal·mol^−1^ and 105.509 kcal·mol^−1^, respectively (Table 3). Both peptides have a stable docking structure with ACE. The 2D and 3D structures of the peptide-ACE complexes are displayed in Figure 5a–d.

There are three main active site pockets in the ACE molecule. S1 pocket has three residues, Ala354, Glu384, and Tyr523. S2 pocket comprises of Gln281, His353, Lys511, His513, and Tyr520. S1′pocket includes residue Glu162 [28,29]. Inhibitory peptides interact with the active pockets of ACE through a variety of forces: electrostatic forces, hydrogen bonds, van der Waals forces, and hydrophobic interactions (Figure 5b,d). Of these forces, hydrogen bonds play a major role. Table 4 shows that FQIN [M(O)] CILR forms twelve hydrogen bonds with ACE residues Asn277, Gln281, Lys511, Tyr523, Ser517, Ser516, Glu123, His353, Glu376. Specifically, FQIN [M(O)] CILR constructs one hydrogen bond with S1 pocket (Tyr523), four bonds with S2 pocket (His353, Lys511, Gln281, His353). TGAPCR forms seven hydrogen bonds with ACE residues Lys511, Gln281, Glu376, Glu384, His353, Tyr394, Arg402. Specifically, Lys511, Gln281 and His353 belong to S2 pocket, link with TGAPCR through three hydrogen bonds and Glu384 belongs to S1 pocket. We speculate that FQIN [M(O)] CILR has a lower IC50 than TGAPCR because FQIN [M(O)] CILR forms a tighter bond with ACE.

### 2.7. Antihypertensive Activity of the Two Peptides on SHRs

**Figure 6:**
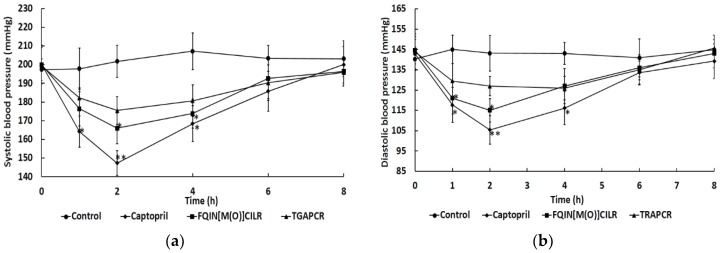
Changes of spontaneously hypertensive rats’ systolic blood pressure (SBP) and diastolic blood pressure (DBP) after oral administration of the two peptides. Saline and Captopril were used as the control and positive control, respectively. Single oral administration was performed with a dose of 10 mg/kg body weight. Blood pressures were measured prior to and 1, 2, 4, 6, 8 h after oral administration. The difference with a value of *p* < 0.05 was considered to be significant. (**a**) SBP changes; (**b**) DBP changes.

The in vivo antihypertensive activity of peptides was assessed by SHRs with a gavage dose of 10 mg/mL. Both FQIN [M(O)] CILR and TGAPCR decreased the SBP of SHRs. FQIN [M(O)] CILR significantly decreased the SBP between 2 to 4 h (*p* < 0.05), with the largest decrease of SBP from 204 to 170 mmHg occurring at 2 h. The SBP then began to recover and maintained a level of 200 mmHg at 8 h (Figure 6a). It was notable that the FQIN [M(O)] CILR reduced the SBP of SHRs by 34 mmHg to the maximum extent. TGAPCR reduced SBP to the lowest point at 2 h. The greatest decline observed was approximately 28 mmHg of SBP from 205 mmHg to 178 mmHg.

In addition, both FQIN [M(O)] CILR and TGAPCR could also affect DBP (Figure 6b). FQIN [M(O)] CILR could significantly reduce the DBP of SHRs from 145 mmHg to 118 mmHg at 2 h (*p* < 0.05), then restore to its original level at 8 h. TGAPCR could decrease DPH to 120 mmHg at 1 h and maintain the DPH until 4 h. TGAPCR can maintain low DBP for a longer period of time compared to FQIN [M(O)] CILR. In general, FQIN [M(O)] CILR has a better antihypertensive effect in SHRs than TGAPCR.

## 3. Discussion

Natural products and derivate are considered relatively safe with limited side effects and thus will become an important source for clinical medications of hypertension in the future [30]. An increasing number of researchers have been investigating natural antihypertensive peptides, in which a variety of antihypertensive peptides with specific sequences and limited side effects have been extracted from animals and plants [10]. Algae accounts for 50% of marine living resources. Thus, marine algae can be used as an important source for extracting active peptides. Researchers have extracted antihypertensive peptides from *Saccharina longicruris* [20], *Palmaria palmate* [21], *Pyropia columbina* [22], and other types of marine algae. However, there are few reports of extracting antihypertensive peptides from *G. lemaneiformis*. Here, we reported that two peptides extracted from *G. lemaneiformis* have the activity to inhibit ACE.

D.Q. Cao et al. have reported that a peptide (QVEY, IC50 = 474.36 μM) isolated from trypsin hydrolyzate of *G. lemaneiformis* had ACE inhibitory activity [31]. FQIN [M(O)] CILR (IC50 = 9.64 ± 0.36 μM) and TGAPCR (IC50 = 23.94 ± 0.82 μM) were also screened from trypsin hydrolyzate of *G. lemaneiformis*, but the two peptides have lower IC50 values than the ACE inhibitory peptide reported above. The two peptides isolated from *G. lemaneiformis* showed higher activity than some reported C-terminal arginine ACE inhibitory peptides YIPIQYVLSR (IC50 = 132.5 μM), YASGR (IC50 = 184 μM) and GNGSGYVSR (IC50 = 29 μM) [32,33]. According to earlier studies, C-terminal arginine plays an important role in inhibiting the activity of ACE. The interaction between C-terminal arginine and ACE can be further analyzed in the molecular docking results.

The properties and stabilities of peptides are prerequisites for the preparation of "functional peptides". According to the information in the Innovagen server, FQIN [M(O)] CILR has poor water solubility. However, in our study, we found that the FQIN [M(O)] CILR had good water solubility when the concentration was lower than 10 mg/mL. The solubility gradually decreased when the concentration was above 10 mg/mL. The hydrophobicity of FQIN [M(O)] CILR is related to the presence of hydrophobic amino acids, such as Phe, Leu, Ile, and Met. The hydrophobicity of FQIN [M(O)] CILR may facilitate the binding of peptides to the hydrophobic active center of ACE, in turn, produce better inhibitory activity.

Small molecule peptides can cross the intestinal wall and enter the blood circulation. Prior to this, peptides must resist the gastrointestinal digestion and maintain their integrity, otherwise their biological activity may be activated or inactivated [34]. Our results suggested that the peptides can maintain the inhibitory activity after being absorbed into the blood.

Angiotensin I-converting enzyme is a carboxypeptidase with very broad substrate specificity [35]. Some ACE inhibitory peptides may become substrates of ACE, which are cleaved into smaller fragments. As a result, the activity of the peptides is altered [34]. For example, ACE hydrolyzed FFGRCVSP isolated from dried bonito to four fragments FF, GR, GV, SP, while the IC50 value increased from 0.4 μM to 4.6 μM [7]. LKPNM (IC50 = 2.4 μM) isolated from dried bonito was hydrolyzed into LKP and NM, and the IC50 value of LKPNM became 1/3 after the pre-incubation with ACE [7]. In view of this, the stability of peptide binding to ACE must be taken into account. Our results proved that FQIN [M(O)] CILR and TGAPCR are stable ACE inhibitors but not substrates of ACE.

There are multiple inhibition patterns of ACE inhibitory peptides: competitive inhibition, noncompetitive inhibition, and mixed-competitive inhibition [36]. The results suggested that both peptides are noncompetitive inhibitors of ACE. Some peptides isolated from food stuffs have also been observed working in noncompetitive inhibition, for example, IFL from fermented soybean food [37], AVKVL from hazelnut [38], and VELWP from cuttlefish [39].

Molecular docking has become an important tool for elucidating the mechanisms of action between ligands and receptors [40]. The results of molecular docking suggested that the two peptides were linked to several pivotal amino acids that are important for ACE. His353, Ala354, and His513 were reported as important residues interacting with Lisinopril which formed six hydrogen bonds with ACE in the absence of the H_2_O molecule [3,28,41]. FQIN [M(O)] CILR and TGAPCR also formed hydrogen bonds with one of the three important residues (His353). Glu384 is the one formed coordination effect with Zn^2+^ [3]. Therefore, TGAPCR may also interfere ACE from combining with Zn^2+^, which in turn may contribute to ACE inhibitory activity. The results also highlighted the important role of arginine in the ACE inhibitory activity of the peptides [32]. The arginine of FQIN [M(O)] CILR formed five hydrogen bonds with the ACE residues Glu376, Asn277, Lys511. ACE residues Glu376, Lys511 and Gln281 formed three hydrogen bonds with the arginine of TGAPCR. Arginine enabled the peptides to form a tight bond with ACE, which facilitated the peptides' inhibitory activity against ACE.

Antihypertensive activity of peptides were further evaluated using spontaneously hypertensive rats (SHRs). The results indicated that captopril resulted in maximal antihypertensive effect on SHRs. Overall, drugs can exert better antihypertensive effects than hydrolysates and antihypertensive peptides derived from foods [2,42]. This may be because antihypertensive drugs have a more stable conformation and a more stringent structure. Both FQIN [M(O)] CILR and TGAPCR decreased the SBP of SHRs. FQIN [M(O)] CILR and TGAPCR reduced the SBP of SHRs by 34 mmHg and 28 mmHg to the maximum extent, respectively. FQIN [M(O)] CILR also decreased the DBP of SHRs significantly (*p* < 0.05). HLFGPPGKKDPV (IC50 = 125 μM), separated from fertilized eggs, had the greatest SBP drop of 30 mmHg, following an oral dose of 10 mg/kg [43]. QPGPT (IC50 = 80.67 μM) and GDIGY (IC50 = 32.56 μM), isolated from jellyfish, reduced the SBP by 13.75 mmHg and 20.67 mmHg, respectively [44]. In comparison to the aforementioned peptides, FQIN [M(O)] CILR (IC50 = 9.64 ± 0.36 μM) may have a strong antihypertensive effect, suggesting that it has considerable potential in the control of blood pressure.

FQIN [M(O)] CILR and TGAPCR are two novel ACE inhibitory peptides, isolated from the trypsin hydrolysate of *G. lemaneiformis*. The results demonstrated that FQIN [M(O)] CILR and TGAPCR not only showed strong ACE inhibitory activity in vitro but also showed strong antihypertensive effects in SHRs. This study suggested that these two natural peptides have the potential to be developed for both antihypertensive drugs and health care products.

## 4. Materials and Methods

### 4.1. Chemicals

ACE (from rabbit lung), hippuryl-histidyl-leucine (HHL), high-performance liquid chromatography (HPLC)-grade acetonitrile (ACN), trifluoroaceticacid (TFA) and porcine pancreatin (8× USP) were purchased from Sigma-Aldrich Co. (St. Louis, MO, USA). Trypsin (EC 3.4.23.4, ≥250. N.F.U/mg solid), pepsin (EC 3.4.23.1, ≥250 U/mg solid), and captopril (>99% purity) were obtained from Solarbio (Beijing, China). All other chemical reagents were of analytical grade.

### 4.2. Preparation of G. Lemaneiformis Hydrolysates

*G. lemaneiformis* was hydrolyzed with 2% trypsin (EC 3.4.23.4, ≥250. N. F. U./mg solid) for 2 h. The 80% alcohol was used to precipitate the impurities in the hydrolysates. The supernatant was concentrated to remove ethanol. Next, the concentrated supernatant was fractionated by ultrafiltration (LNG-UF-101, Laungy Membrane Filtration Technology Co. Ltd., Shanghai, China) and the fraction (<3 KDa) was collected and freeze-dried before storage at −80 °C.

### 4.3. Identification of Peptides by LC-MS/MS

Peptide sequences were determined according to Kai Lin [45]. Identification of peptides in 3 KDa permeates was performed using a Q-Exactive mass spectrometer (Thermo Fisher Scientific, Waltham, MA, USA) coupled with a Thermo Scientific EASY-nLC 1000 System (Thermo Fisher Scientific, Waltham, MA, USA). Samples were loaded in a reverse-phase trap column (2 cm × 100 μm, 5 μm-C18), which was connected to a reverse-phase analytical column (75 μm × 100 μm, 3 μm-C18). The purified sample was injected into the trapping column at a flow rate of 300 nL/min. The mass spectrometer (MS) was operated in positive-ion detection mode, and the most abundant precursor ions from the scanning range of 300−1800 *m*/*z* were selected to obtain MS data. Peptide sequences were determined based on the MS/MS spectra and Mascot 2.2 (Matrix Science Inc., Boston, MA, USA) searches of dataset of *G. lemaneiformis* (Accession: SRX258772, download from SRA database of NCBI).

### 4.4. Synthesis of ACE Inhibitory Peptides

The inhibitory peptides were chemically synthesized in Shanghai Qiangyao Biotechnology Co., Ltd. (Shanghai, China). The peptides were synthesized using the Fmoc solid-phase method. To increase the stability of FQINMCILR, Methionine is selectively modified to methionine sulfoxide (FQIN [M(O)] CILR). The purity of the two peptides is 97.85% and 97.59%.

### 4.5. Measurement of ACE Inhibition Activity

ACE inhibition activity was assayed according to Cushman and Cheung, with some modification [27]. ACE was dissolved in sodium borate buffer (pH 8.3, containing 0.3 M sodium chloride) to 0.1 U/mL for the assay. Synthetic peptides were dissolved in distilled water to seven concentration levels. Then, 20 μL of a certain concentration of peptide was mixed with 10 μL ACE solution. The mixture was incubated at 37 °C for 5 min, and then 50 μL of 5 mM HHL (sodium borate buffer pH 8.3, containing 0.3 M sodium chloride) was added to the above mixture to start the reaction. The reaction was maintained at 37 °C for 60 min, and then 150 μL of 1 M HCl was added to stop the reaction. The solution was filtered through a 0.22 micron membrane. Next, 20 μL of reaction solution was injected into a RP-HPLC (Shimadzu, Kyoto, Japan) fixed with Eclipse XDB-C18 column (4.6 mm × 150 mm × 5 μm, Agilent Technologies Inc., St. Clara, CA, USA) to measure the concentration of hippuric acid (HA). The absorbance was detected at 228 nm. All determinations were triplicate. The activity of ACE inhibition was calculated as followed:

ACE inhibitory activity (%) = (A_control_ − A_inhibitor_)/A_control_ × 100

where A_inhibitor_ is the relative area of the hippuric acid (HA) peak obtained from the reaction of ACE and HHL with inhibitor. A_control_ is the relative area of the hippuric acid (HA) peak obtained from the reaction of ACE and HHL without inhibitor. IC50 is defined as the concentration of peptides that can inhibit half of the ACE activity.

### 4.6. Properties of Peptides

The properties of ACE inhibitory peptides are important for future research. These parameters can be retrieved from the online database. The ToxinPred server can analyze toxicity of the peptides (http://crdd.osdd.net/raghava/toxinpred/). The online Innovagen server was used to evaluate the solubility of the peptide (www.innovagen.com/proteomics-tools).

### 4.7. Stability Study for ACE Inhibitory Activity

#### 4.7.1. Thermal Stability for Peptides

The synthetic peptide solutions (0.5 mg/mL) were incubated at different temperatures (0 °C, 20 °C, 40 °C, 60 °C, 80 °C, 100 °C) for 2 h. Next, 20 μL of peptide was used to assay the ACE inhibitory activity using the above method [39].

#### 4.7.2. pH Stability for Peptides

The synthetic peptide solutions (0.5 mg/mL) were incubated at different pH levels (2, 4, 6, 8, 10, 12) for 2 h, and then solutions were neutralized to pH 7.0 [39]. Then, 20 μL of peptide was used to assay the ACE inhibitory activity using the above method.

#### 4.7.3. Gastrointestinal Stability of Peptides

Gastrointestinal stability of peptides was evaluated in vitro [46,47]. Peptides were dissolved in a 0.1 M KCl-HCl buffer (pH 2.0) to the concentration of 0.1 mg/mL and 0.5 mg/mL. Pepsin (≥250 U/mg) was added to a peptide solution to the final concentration 0.8 mg/mL. The solution was then incubated at 37 °C for 4 h. The pepsin was inactivated through boiling the solution for 10 min. The pH of the solution was adjusted to 7.0 with 1 M NaOH. The solution was centrifuged at 12,000 rpm for 5 min and the supernatant (20 μL) was taken out to determine the ACE inhibitory activity. The remaining supernatant was further incubated with pancreatin (10 mg/mL, 8× USP) at 37 °C for 4 h. The reaction was stopped by boiling the solution for 10 min. The solution was centrifuged at 12,000 rpm for 10 min, and then the supernatant (20 μL) was used to detect ACE inhibitory activity.

### 4.8. Stability of Peptides against ACE

The stability of peptides against ACE was assayed according to Salampessy. J and Fujita. H [48,49]. 30 μL of peptides (0.1 mg/mL) reacted with 30 μL 0.1 U/mL ACE solution at 37 °C for 24 h. The ACE was then inactivated through boiling the solution for 10 min. Then, 20 μL of peptides were used to detect ACE inhibitory activity in the above method.

### 4.9. Determination of Inhibitory Pattern

Peptides were dissolved in distilled water to the concentration of 1 mg/mL and 0.5 mg/mL, and HHL was dissolved to the concentration of 4 mg/mL, 2 mg/mL, 1 mg/mL and 0.5 mg/mL. Different concentrations of the peptide were reacted at different concentrations of HHL. Then, 20 μL of peptide solution was used to determine the ACE inhibitory activity using the above method. Lineweaver—Burk plots were applied to confirm the ACE inhibitory pattern of peptide. The inhibitory constant (Ki) was the intercept of the *X*-axis of the plot, of which the *Y*-axis displayed the slopes of Lineweaver-Burk line and the *X*-axis indicted peptides concentrations [25].

### 4.10. Molecular Docking

The affinity of peptides to inhibit ACE was simulated and evaluated by Discovery Studio 3.5 (DS 3.5, Accelrys, San Diego, CA, USA), according to the reported method with some modification [32]. In the docking experiments, the crystal structure of human tACE (PDB ID: 1O8A, http://www.rcsb.org/pdb/explore/explore.do?structureId=1O8A) was as receptor. The 3D structure of the peptides was designed by Discovery Studio 3.5 (DS 3.5, Accelrys, San Diego, CA, USA). Then the peptide was protonated at pH 7.0 and energetically minimized by the CHARMm force field. The structure of ACE was removed water, cleaned protein and added hydrogen. The CDOCKER was selected to simulate the docking of receptors and peptide ligands. The binding site sphere was *x*: 40.302, *y*: 37.243 and *z*: 48.948. Rigid residues were residues within the sphere with a 20 Å radius and with zinc as the center. CDOCKER module uses a CHARMm-based molecular dynamics (MD) scheme to dock ligands into a receptor binding site. Random ligand conformations are generated using high-temperature MD. The conformations are then translated into the binding site. Candidate poses are then created using random rigid-body rotations followed by simulated annealing. A final minimization is used to refine the ligand poses. -CDOCKER ENERGY and -CDCKER INTERACTION ENERGY are two criteria for evaluating CDOCKER results. -CDOCKER ENERGY indicates the negative of the total energy. -CDOCKER INTERACTION ENERGY indicates the negative of the interaction energy. The optimal conformation of peptide-ACE complex has the highest values of -CDOCKER ENERGY and -CDOCKER INTERACTION ENERGY.

### 4.11. Antihypertensive Effect on SHRs

Spontaneously hypertensive rats (SHRs) (male, 10-week-old, 250–300 g body weight, specific, pathogen-free) were bought from Vital River Laboratory Animal Co., Ltd. (Beijing, China). Animals used in this study were maintained in accordance with the guidelines of the Institutional Research Council’s Guide for the Care and Use of Laboratory Animals. SHRs were housed under 12 h day/night cycle at 22 ± 2 °C and fed with tap water and a standard diet ad libitum. The SHRs were randomly separated into a positive group, control group, and two experiment groups. There were six SHRs in each group. Before the formal start of the test, SHRs had to adapt to measuring the environment in advance. Once the tail systolic blood pressure (SBP) rates were above 180 mmHg, the gavage administration could start.

Normal saline was the solvent to dissolve peptides and control reagent. Captopril (99% purity, Solarbio Technology Co., Ltd., Beijing, China) was the positive group. The same volume of normal saline was the control group. Tail-cuff method was performed for systolic blood pressure (SBP) and diastolic blood pressure (DBP) measurements with a non-invasive CODA device (Kent Scientific Co., Torrington, CT, USA). The blood pressure was measured in 0, 1, 2, 4, 6, 8 h after gavage administration. Measurements were repeated three times at each time point.

### 4.12. Statistical Analysis

Data analysis was performed using SPSS Version 17.0 (SPSS Inc., Chicago, IL, USA). To compare the mean differences among the groups, one-way analysis of variance was used. The results are shown as the mean ± SD, and *p* < 0.05 was considered to be statistically significant.

## 5. Conclusions

Two ACE inhibitory peptides, identified as FQIN [M(O)] CILR and TGAPCR from *G. lemaneiformis*, inhibit ACE in a noncompetitive pattern. Molecular docking results showed that the peptides were mainly linked to ACE via hydrogen bonds and produce inhibitory activity. Animal experiment has shown that FQIN [M(O)] CILR and TGAPCR can reduce blood pressure of SHRs. Therefore, these peptides have the potential to treat hypertension, while *G. lemaneiformis* can be developed as an antihypertensive food.

## Figures and Tables

**Table 1:** Physicochemical characteristics of the two peptides.

Peptides	Number of Residues	Molecular Weight (Da)	ToxiPed	Solubility in Water	Iso-Electric Point (PI)
FQIN [M(O)] CILR	9	1153.43	non-toxin	poor	8.61
TGAPCR	6	603.69	non-toxin	good	8.55

**Table 2:** Kinetics parameters of ACE-catalyzed reactions in different peptide concentrations.

Kinetics Parameters	Control	FQIN [M(O)] CILR		TGAPCR	
		1 mg/mL	0.5 mg/mL	1 mg/mL	0.5 mg/mL
Km (mM)	2.66 ± 0.21	2.66 ± 0.21		2.66 ± 021	
Vmax (mg^−1^·mL·min)	2.23 ± 0.67	0.10 ± 0.05	0.14 ± 0.02	0.11 ± 0.03	0.17 ± 0.02
Ki (mM)		0.71 ± 0.04		0.86 ± 0.06	

**Table 3:** Docking energies for optimal conformation of two ACE inhibit peptides and ACE.

Peptides	-CDOCKER ENERGY (kcal·mol^−1^)	-CDOCKER INTERACTION ENERGY (kcal·mol^−1^)
FQIN [M(O)] CILR	158.117	145.01
TGAPCR	90.4226	105.509

**Table 4:** Hydrogen bonds formed between two ACE inhibitory peptides and ACE.

Peptides	Donor Atom	Acceptor Atom	Distance (Å)	Active Pocket
FQIN [M(O)] CILR	A: ASN277: HD22	F: O125	2.39	
	A: GLN281: HE22	F: O89	1.97	S2
	A: LYS511: HZ1	F: O89	2.02	S2
	A: LYS511: HZ3	F: O126	2.31	S2
	A: TYR523: HH	F: S63	2.24	S1
	F: H145	A: SER517: O	1.95	
	F: H146	A: SER516: O	1.98	
	F: H148	A: GLU123: OE1	2.14	
	F: H158	A: HIS353: NE2	2.33	S2
	F: H161	A: GLU376: OE1	2.44	
	F: H161	A: GLU: 376: OE2	2.03	
	F: H163	A: GLU: 376: OE1	2.32	
TGAPCR	A: GLN281: HE22	T: O2	2.06	S2
	A: LYS511: HZ1	T: O2	1.78	S2
	T: H71	A: GLU376: OE2	2.16	
	T: H75	A: GLU384: OE2	2.26	S1
	T: H76	A: HIS353: NE2	2.24	S2
	T: H78	A: TYR394: OH	2.32	
	T: H79	A: ARG402: O	2.41	

F is an abbreviation of FQIN [M(O)] CILR; T is an abbreviation of TGAPCR.
